# Sleep-Specific Processing of Auditory Stimuli Is Reflected by Alpha and Sigma Oscillations

**DOI:** 10.1523/JNEUROSCI.1889-21.2022

**Published:** 2022-06-08

**Authors:** Malgorzata Wislowska, Wolfgang Klimesch, Ole Jensen, Christine Blume, Manuel Schabus

**Affiliations:** ^1^Centre for Cognitive Neuroscience, University of Salzburg, Salzburg, 5020, Austria; ^2^Laboratory for Sleep, Cognition and Consciousness Research, University of Salzburg, 5020, Salzburg, Austria; ^3^Centre for Human Brain Health, University of Birmingham, Birmingham, B12 2TT, United Kingdom; ^4^Centre for Chronobiology, Psychiatric Hospital of the University of Basel, Basel, CH-4002, Switzerland; ^5^Transfaculty Research Platform Molecular and Cognitive Neuroscience, University of Basel, Basel, 4055, Switzerland

**Keywords:** auditory, brain oscillations, EEG, information processing, MEG, sleep

## Abstract

Recent research revealed a surprisingly large range of cognitive operations to be preserved during sleep in humans. The new challenge is therefore to understand functions and mechanisms of processes, which so far have been mainly investigated in awake subjects. The current study focuses on dynamic changes of brain oscillations and connectivity patterns in response to environmental stimulation during non-REM sleep. Our results indicate that aurally presented names were processed and neuronally differentiated across the wake-sleep spectrum. Simultaneously recorded EEG and MEG signals revealed two distinct clusters of oscillatory power increase in response to the stimuli: (1) vigilance state-independent θ synchronization occurring immediately after stimulus onset, followed by (2) sleep-specific α/σ synchronization peaking after stimulus offset. We discuss the possible role of θ, α, and σ oscillations during non-REM sleep, and work toward a unified theory of brain rhythms and their functions during sleep.

**SIGNIFICANCE STATEMENT** Previous research has revealed (residual) capacity of the sleeping human brain to interact with the environment. How sensory processing is realized by the neural assemblies in different stages of sleep is however unclear. To tackle this question, we examined simultaneously recorded MEG and EEG data. We discuss the possible role of θ, α, and σ oscillations during non-REM sleep. In contrast to versatile θ band response that reflected early stimulus processing step, succeeding α and σ band activity was sensitive to the saliency of the incoming information, and contingent on the sleep stage. Our findings suggest that the specific reorganization of mechanisms involved in later stages of sensory processing takes place upon falling asleep.

## Introduction

Sleep differs from wakefulness at behavioral, cognitive, and neuronal levels. Nonetheless, several recent studies documented the capacity of the brain to interact with (usually auditory) external cues even during consolidated stages of sleep. According to these studies, the sleeping brain can detect novelty (Ruby et al., 2008), discriminate stimuli on a semantic (Perrin, 1999) and lexical (Kouider et al., 2014) level, distinguish familiarity (Blume et al., 2018) and emotional tone (del Giudice et al., 2016b), or even track continuous speech (Legendre et al., 2019). These processes endure despite a major reorganization of brain activity patterns during sleep. This is an interesting observation because the relationship between oscillations and cognitive processes has been, so far, investigated mainly in awake subjects.

In the current project, we therefore focused on the interplay between oscillatory activity and cognitive processes during sleep. For this purpose, we stimulated participants with auditory cues and compared brain responses across wakefulness and the different stages of non-REM (NREM) sleep. We used stimuli of a high personal relevance, including the subject's own name spoken by a close family member. This way, we increased the bottom-up stimulus strength, which ensues from subjective importance and frequent exposure across lifetime (Holeckova et al., 2006). Own names and familiar voices elicit a distinct brain response even in patients whose level of consciousness diminished after a severe brain injury (Bekinschtein et al., 2004; Perrin et al., 2006; del Giudice et al., 2016a). Preserved processing of names and voices has likewise been observed in participants falling asleep (Perrin et al., 1999; Portas et al., 2000; Blume et al., 2018), rendering these specific stimuli pertinent for studying information processing during sleep. Consequently, we investigated how the oscillatory responses to aurally presented names change as the brain progresses through different stages of NREM sleep. Thereupon, we deliberate on the possible functional meaning of the observed brain dynamics.

Over the last century that followed the legendary first recording of α rhythms in the brain (Berger, 1929), a multitude of research drew a link between brain oscillations and cognition (Varela et al., 2001; Buzsáki and Draguhn, 2004). Even consciousness has directly been related to the activity of synchronized neural networks (Crick and Koch, 2003; Owen and Guta, 2019), and oscillations have been discussed to orchestrate information transmission (Sauseng and Klimesch, 2008; Siegel et al., 2012). A hierarchy of nonoverlapping and distinct frequency bands allows executing various cognitive tasks in exact temporal order (Pletzer et al., 2010; Klimesch, 2012). For example, the θ oscillation (∼4–7 Hz) has been associated with working and episodic memory (Doppelmayr et al., 1998; Jensen and Tesche, 2002), whereas α (∼8–12 Hz) has been related to semantic memory (Klimesch et al., 1997; Fellinger et al., 2012), attention (Klimesch, 1999; Thut et al., 2006), or disengagement of irrelevant cortical regions (Jokisch and Jensen, 2007; Haegens et al., 2010). In sleep, the function of these oscillations is, however, much less investigated and conclusive. The major contributions for the functional interpretation of oscillations in sleep come from the field of overnight memory consolidation in humans, where slow-wave (∼0.5–1 Hz) (Marshall et al., 2006), θ (∼4–7 Hz) (Schreiner et al., 2018), and spindle (∼11–15 Hz) (Cairney et al., 2018) frequencies play the central role. Yet cognitive processes that appear to persist during sleep are, of course, not limited to internal memory reorganization.

Overall, we lack a systematic understanding of the neuronal underpinnings of the cognitive processes still being intact during sleep. Therefore, in the current MEG/EEG study, we aimed to answer the following questions: (1) to which degree the sleeping brain is still capable of processing environmental stimuli, (2) which oscillatory mechanisms govern that processing, and (3) how connectivity patterns change across the continuum from wake to NREM sleep.

## Materials and Methods

The study was approved by the University of Salzburg local ethics committee and conducted in accordance with the Declaration of Helsinki. Written informed consent was obtained from all research participants before inclusion. Volunteers received financial or course credit compensation for their time.

### Participants

Twenty-nine young, healthy, right-handed, native German speakers participated in the experiment. All were nonsmokers and had no record of a neurologic, psychiatric, or sleep disorder. Two participants were later excluded because of technical problems during data acquisition. Ultimately, we analyzed data from 27 subjects (16 females) with an average age of 24.93 (SD = 2.37) years, of which 11 reached stable deep sleep.

### Experimental design

**Figure 1.**
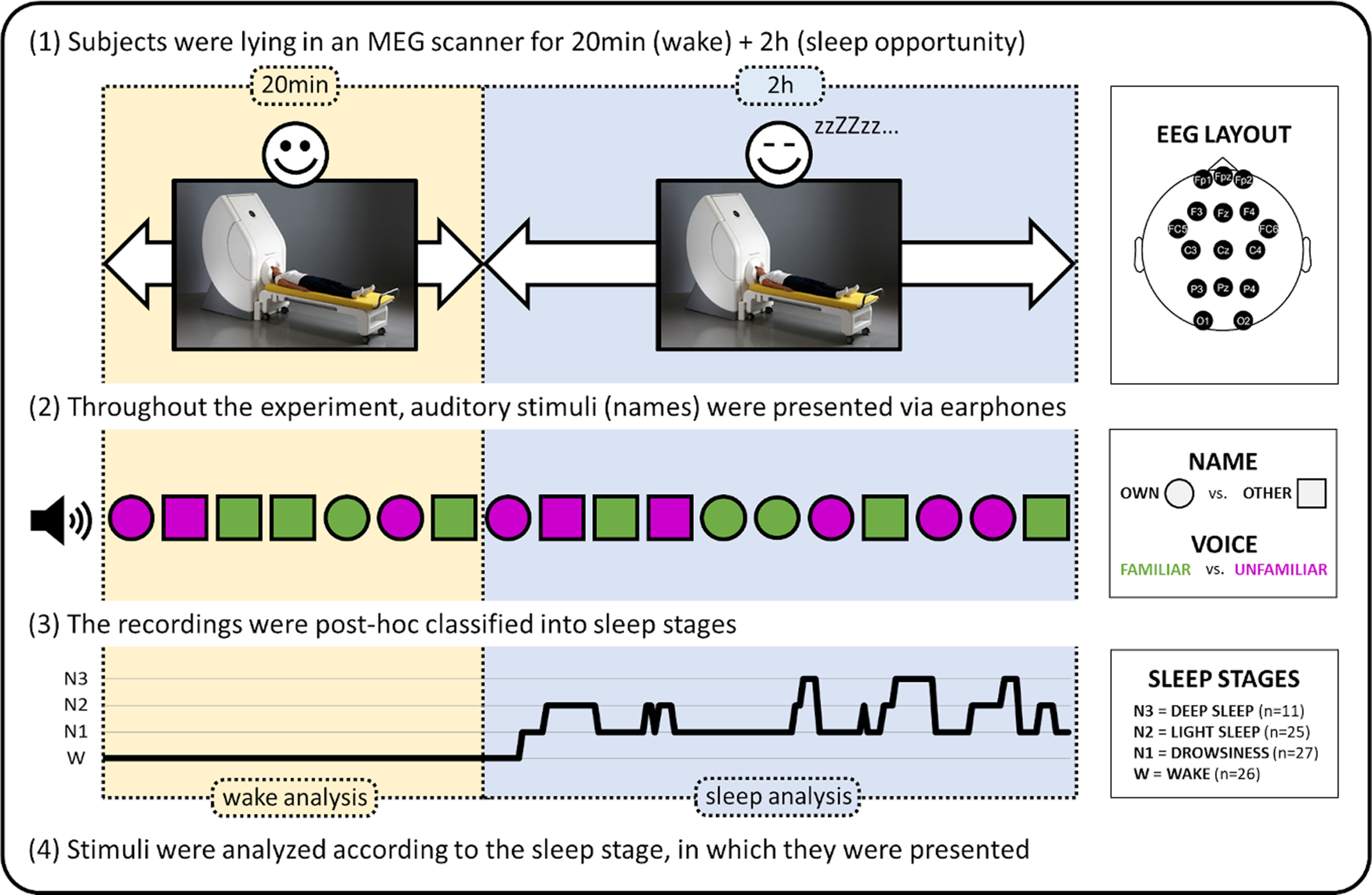
Study design. Processing steps from MEG/EEG acquisition to analysis of the data are illustrated. Spoken first names were presented to the participants lying in the MEG scanner. Volunteers were requested to stay awake with eyes open for 20 min, and then close their eyes and try to sleep for another 2 h. Stimuli were presented aurally and in a pseudo-random order every 2.5 to 6 s. For the analysis, stimuli were grouped according to (1) the name type, (2) the uttering (familiar or unfamiliar) voice, and (3) the current sleep stage at stimulus presentation.

Participants visited the laboratory 1 week before the MEG session. They assisted in creating their individual stimulus set by selecting unfamiliar names and getting instructed on how to record familiar voice audio files. Thereafter, subjects received a wrist-actigraphy (Cambridge Neurotechnology Actiwatch), which they were wearing on their left (nondominant) hand for the next 7 d. Afterward, participants returned to the laboratory for the MEG session. The recorded actigraphy data helped us to control for adherence to the study protocol. Subjects were instructed to keep a regular sleep-wake cycle (and sleep ∼8 h each night) between the two laboratory visits, except for the last night, when they were asked to restrict their sleep time to 6 h. On the day of the experimental visit, the volunteers reported to the MEG laboratory (14 subjects at around 9:00 A.M., 13 subjects at around noon), where they were familiarized with the protocol. Upon signing the informed consent, the subjects had to change into scrubs and remove all metallic parts from their body. After fixing localization coils and polysomnography channels, participants entered the magnetically shielded room and lay down in a supine position on an MEG-compatible bed. We maximized the comfort with individually adjusted pillows and blankets, and provided participants with earphones. A 5 min rest recording (not reported here) was followed by the main experiment that consisted of 20 min wake session and a 2 h sleep opportunity session (Fig. 1). Throughout the entire 2h20min of the experiment participants were played an auditory stream of first names. There was no specific task instruction for participants, except of remaining awake with eyes open during the wake part, and there being the opportunity to fall asleep with eyes closed during the sleep part. For the analysis of brain activity during wakefulness, only data from the first stable 20 min wake session with eyes-open were included.

### Stimuli

We adopted a passive version of a previously established “own name paradigm” (Perrin et al., 2005; Fellinger et al., 2011; del Giudice et al., 2016b), where the first name of a subject is presented among other first names. During their first visit in the laboratory, participants were presented with a list of common Austrian first names, matched with their own first name in terms of the syllable number, gender, and likelihood of occurrence in the general population. From this list, participants selected two names with no strong personal emotional valence. The subject-specific set of three names (subject own name and two other names) was recorded by two speakers of the same gender: first was a person closely related to the participant (e.g., parent, partner, a close friend; familiar voice condition), second was an unfamiliar, dialect-free native German speaker (unfamiliar voice condition). The recordings of the audio files were matched in length, as much as possible, while preserving their natural tone. All stimuli were preprocessed (denoised, normalized and filtered) using Audacity software (http://audacityteam.org/). Accordingly, a 2 × 2 design was created, with two types of names (own and other) and two types of voices (familiar and unfamiliar). Stimuli on average lasted 725 ms (1 SD = 0.164 ms). During the MEG session, the names were presented via MEG-compatible pneumatic earphones (SOUNDPIxx, VPixx Technologies) with interstimulus interval pseudo-randomly varying between 2.5 and 6 s (in 500 ms steps). As in earlier studies (e.g., Ameen et al., 2022), loudness of the stimuli was adjusted for each participant individually and based on a subjective feedback, aiming at the volume clearly audible, which at the same time allows falling asleep. Consequently, the volume level of the presented stimuli varied between 61 and 84 dB. After preprocessing (see below), the following average number of trials per subject remained for analyses: 60 (±13) trials during wake (W), 93 (±70) trials during drowsiness (N1), 130 (±90) trials during light sleep (N2), and 42 (±31) trials during deep sleep (N3).

### Data acquisition

Brain and peripheral signals were recorded at 1000 Hz with hardware filters between 0.1 and 330 Hz, in a passive magnetically shielded room using a whole head MEG (Elekta Neuromag Triux). Magnetic brain data were sampled with 204 orthogonal gradiometers and 102 magnetometers. Simultaneously, we recorded electric brain signal with 18 active monopolar EEG channels spanning the whole scalp according to the international 10–20 system (Fp1, Fpz, Fp2, F3, Fz, F4, FC5, FC6, C3, Cz, C4, P3, Pz, P4, O1, O2, left and right mastoid), with a reference placed on the left ear and a ground placed on the right shoulder. Additional bipolar peripheral channels were recorded: EMG on the chin, ECG across the chest, and two EOG (horizontal and vertical), following standard recommendations for sleep EEG data acquisition of the American Association of Sleep Medicine (Iber and Iber, 2007). EEG impedances were kept <5 kOhm for the monopolar channels, and <75 kOhm for the bipolar channels. The position of the head in the MEG helmet was acquired at the beginning of each session with 5 HPI localization coils (three placed on the forehead, two placed on the left and right periauricular points). Shape of the head as well as positions of the electrodes and the localization coils were 3D digitized with Polhemous FASTTRACK.

### Sleep analysis

PSG recordings were *post hoc* automatically staged (Somnolyzer 24 × 7, Koninklijke Philips) (Anderer et al., 2005, 2010) and visually controlled by an expert from The Siesta Group, according to current standard criteria (Iber and Iber, 2007).

#### Arousals

From sleep analysis, we excluded trials containing signs of cortical arousal. An automatized MATLAB script (Jagannathan et al., 2018) classified 4-s-long epochs (from −2 to 2 s around stimulus onset) according to Hori staging (Hori, 1990; Tanaka et al., 1996; Goupil and Bekinschtein, 2012). For the classification, we used the entire set of 16 EEG scalp electrodes, with the signal downsampled to 250 Hz and filtered between 1 and 30 Hz. Epochs labeled as “Alert” (Hori Stages 1 and 2) were subsequently removed from all analyses.

### Data preprocessing

MEG and EEG data were analyzed using MATLAB (version 2017a) and the Fieldtrip toolbox (Oostenveld et al., 2011). The signal was high-pass filtered at 0.5 Hz. EEG was rereferenced to mastoid electrodes (A1 and/or A2), selected depending on the visually inspected and best signal quality. We identified and removed independent components corresponding to eye-blinks (during wake) and heart-beat (during wake and sleep) and projected out exogenous noise with a signal-space-projection algorithm **(**Uusitalo and Ilmoniemi, 1997) (MATLAB implementation: https://gitlab.com/obob/obob_ownft, obob_apply_ssp function). Further artifacts and noisy channels were semiautomatically identified in 1 s segments. Finally, continuous data (after independent components analysis and signal-space-projection) was downsampled to 512 Hz, filtered <250 Hz, and segmented into trials from −2.3 to 2.3 s relative to stimulus onset (longer trials were used to avoid the boundary effect disturbing data of interest in the time-frequency analysis). Trials containing noisy 1 s segments were excluded. In the final step, an additional data quality assurance was performed visually on each individual recording. For the sensor level analysis, we interpolated missing MEG sensors.

### Data analysis at the sensor level

#### Evoked brain responses

For the time-locked analysis, we averaged across trials filtered <40 Hz and calculated the difference from power in the prestimulus baseline between −0.5 and −0.1 s (absolute baseline in FieldTrip). For the MEG analysis, magnetic fields of the corresponding planar gradiometers were combined (root of sum of squares). Last, a linear trend was removed from windows of interest (spanning −0.5 to 1.5 s relative to stimulus onset). In the final step, the individual evoked responses were averaged across subjects.

#### Induced brain responses

For the induced response analysis, we used a sliding window approach on the single-trial demeaned data. Data segments were extracted every 50 ms and multiplied with a Hanning taper. Phase and power of frequencies between 1 and 30 Hz were calculated in 1 Hz steps using a frequency-dependent time window length of 3 cycles (except for frequencies <3 Hz, where we used only 1 cycle for convenience). For the MEG analysis, oscillatory power of the corresponding planar gradiometers was combined (root of sum of squares). Finally, we averaged across trials, cut windows of interest (spanning from −0.5 to 1.5 s), and averaged across subjects.

#### Auditory ROI

In the MEG data, auditory ROIs were identified at single-subject level and based on evoked response in the 0.05-0.5 s time window (across stimuli). Fifteen combined gradiometers with the highest power relative to the baseline (−0.5 to −0.1 s before stimulus onset) were then selected. For the EEG analysis, always the same six sensors were used: FC5, C3, P3, FC6, C4, and P4.

#### Sleep spindles

Sleep spindles were detected automatically at EEG electrodes C3 and C4 during sleep stages N2 and N3 with a two-stage algorithm (ASK analyzer, The Siesta Group). First, possible spindles were identified (Schimicek et al., 1994) in data filtered between 11 and 16 Hz, based on the following criteria (Broughton et al., 1978; Schimicek et al., 1994; Anderer et al., 2005): (1) amplitude >12 μV and (2) duration between 0.3 and 2 s. Second, a linear discriminant analysis, previously trained on visually scored spindles, was run on the possible spindles. In our analyses, we used spindle events of frequency between 11 and 15 Hz and with a discriminant score >0.8, corresponding to a sensitivity of ∼90% (Diekelmann and Born, 2010). Sleep spindles that occurred in an overlapping time window at C3 and C4 were considered to represent the same spindle event.

#### Oscillations generating evoked responses

The analysis of oscillations contributing to the generation of the ERPs was performed on the grand-average EEG time-locked data (i.e., broadband signal, filtered between 0.5 and 40 Hz, as explained in the previous sections). The ERPs were filtered with 8 different bandpass FIR filters, using the following cutoff values: 1-2 Hz (δ), 4-7 Hz (θ), 8-12 Hz (α), 8-10 Hz (lower α), 10-12 Hz (upper α), 11-15 Hz (σ), 11-13 Hz (slow σ), and 13-15 Hz (fast σ). Peaks of the broadband and each narrow-band signal were detected with MATLAB's *findpeaks.m* routine. Finally, peaks of the broadband signal were compared with the peaks of each narrow-band signal and classified as aligned, when they occurred in the same time point (±3 sample points).

### Statistical analysis

We tested for statistical differences in time-frequency spectra between conditions of interest (two types of name and two types of voice) in each sleep stage separately, as well as between sleep stages, using nonparametric cluster-based permutation statistics (Maris and Oostenveld, 2007), which accounts for the multiple-comparison problem. We used a two-sided test for dependent samples (*depsamlesT* in Fieldtrip), with the α level value set to 2.5%. The histogram of test statistics was built based on 1000 permutations. The same analysis was performed for prestimulus to poststimulus contrasts. For the *prestimulus* condition, data between −0.5 and −0.1 s before the name onset was averaged for each time, frequency, sensor, and subject separately. The averaged values were then repeated over time window of the same length as the *poststimulus* interval (0-1.5 s relative to the stimulus onset).

Distributions of sleep spindle features (number and amplitude) for the two name types were statistically quantified with a two-sample Kolmogorov–Smirnov test.

### Source reconstruction

In the last step, we reconstructed sources underlying the observed sensor level results. For the head modeling, we were able to acquire individual structural MRI scans from 9 subjects; for the rest, we used a standard template brain of the MNI. The brain anatomy of each subject was approximated with a single shell model. A template brain was discretized into a grid of 1.5 cm resolution and then wrapped to match each individual head model (canonical mesh).

We reconstructed single trials at each source grid point with adaptive spatial filters (LCMV beamformers) (Van Veen et al., 1997), using filters built on data from all conditions, separately for each sleep stage, and filtered <40 Hz. We integrated the signal recorded with gradiometers and magnetometers. To estimate generators of the specific frequency responses, we decomposed the source level signal into the time-frequency domain with the exact same parameters as for the sensor level analysis. Contrasts of interest (names or voices) were calculated for each subject individually, and the results were averaged across subjects.

### Code accessibility

The code used for data analysis is available at the first author's Gitlab repository (https://gitlab.com/Wislowska/fSON_MEG_project).

## Results

### The sleeping brain detects and discriminates environmental cues

Overall, results reveal that the brain can detect auditory stimuli in wakefulness as well as sleep. Interestingly, more complex stimulus discrimination persists across all stages of NREM sleep. It should be noted that, in this study, stable deep (N3) sleep was reached by 11 of the 27 subjects as we refrained from strict sleep restriction the night before to get an estimate of natural sleep in the MEG as much as possible.

#### Stimulus detection

**Figure 2.**
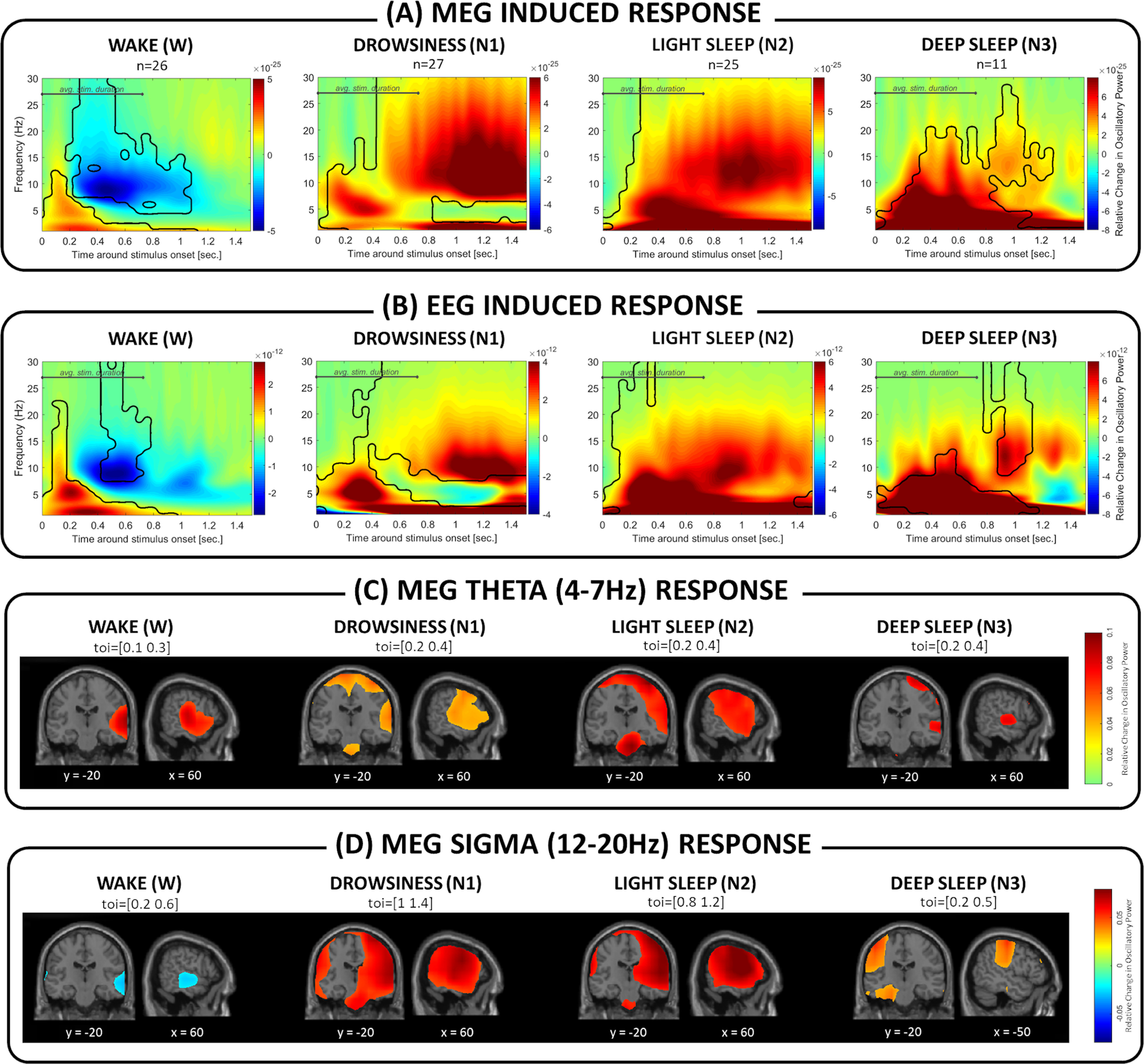
Oscillatory power response to auditory stimuli presented in various stages of NREM sleep. In (***A***) depicting MEG sensors and (***B***) depicting EEG sensors, NREM sleep is characterized by synchronization within the α/σ band (∼9-16 Hz) and a lack of α band (∼8-12 Hz) desynchronization. The plots are normalized against absolute baseline between 0.5 and 0.1s before stimulus onset. Areas outlined in black represent significant clusters for prestimulus versus poststimulus contrasts. ***C***, ***D***, Source level topographical distribution of the MEG θ (4-7 Hz) and σ (12-20 Hz) frequency response, respectively. Primary auditory areas contribute to the θ response in wakefulness and in NREM sleep, along with coactivation in sensorimotor areas and the brainstem after sleep onset. Sources of σ modulation in light sleep (N1 and N2) included thalamus, auditory, and sensorimotor areas, while in deep sleep σ was generated in sensorimotor areas, frontal eye field, and parahippocampal regions. Maps represent the highest 60% of the difference between baseline and poststimulus intervals, where *y* and *x* coordinates indicate locations in MNI space. Toi = time of interest.

As shown in Figure 2, auditory stimuli modulated ongoing brain activity in wakefulness as well as NREM sleep, when measured with MEG (Fig. 2*A*) and EEG (Fig. 2*B*) sensors. Stimulus-induced oscillatory power (across a broad frequency spectrum) was significantly different from prestimulus baseline in wakefulness (MEG: p_cluster1_ = <0.001, p_cluster2_ = 0.004; EEG: p_cluster1_ = <0.001, p_cluster2_ = 0.007), as well as all stages of NREM sleep: drowsiness (MEG: p_cluster_<0.001; EEG: p_cluster_<0.001), N2 (MEG: p_cluster_<0.001; EEG: p_cluster_<0.001) and deep sleep (MEG: p_cluster_ = 0.002; EEG: p_cluster1_ = 0.003, p_cluster2_ = 0.02).

Frequency-resolved MEG (Fig. 2*A*) and EEG (Fig. 2*B*) responses during wakefulness displayed a typical profile, with early δ/θ (∼1-7 Hz) synchronization, followed by α (∼8-12 Hz) desynchronization. θ synchronization persisted across all stages of NREM sleep (Fig. 2*A*,*B*). α-frequency desynchronization, on the other hand, was exclusively observed during wakefulness. Furthermore, oscillations within the broad α/σ (∼8-20 Hz) range synchronized at ∼700 ms after stimulus, in light (N1 and N2), and to a smaller extent in N3.

To estimate the sources within the brain that underlie the observed oscillatory activity, we applied a beamformer technique to the MEG signal. As depicted in Figure 2*C*, generators of the θ activity were identified in the primary auditory cortices (Brodmann area [BA] 41), independently of vigilance state (*xyz* coordinates of the source level response in the MNI space: W = [70, −21, 10]; N1 = [64, −22, 12]; N2 = [66, −10, 8]; N3 = [59, −14, 4]). During NREM sleep, additional sources included the sensorimotor areas (BA 1, 4, 6; *xyz* coordinates: N1 = [37, −22, 68]; N2 = [55, −6, 9], N2 = [41, −10, 65]; N3 = [59, −10, 38]) and the brainstem (*xyz* coordinates: N1 = [9, −21, −38]; N2 = [8, −18, −34]; N3 = [−6, −24, −44]).

Sleep-specific α/σ power increase had similar generating sources in N1 and N2 sleep, including: primary auditory areas (BA 41; *xyz* coordinates: N1 = [53, −19, 6]; N2 = [55, −19, 9]), Wernicke's area (BA 40; *xyz* coordinates: N1 = [55, −28, 38]; N2 = [59, −23, 37]), sensorimotor areas (BA 1, 4, 6, 7; *xyz* coordinates: N1 = [50, −22, 54], [56, −11, 37], [39, −19, 65], [25, −70, 54]; N2 = [61, −3, 18], [26, 3, 64], [12, −61, 68], [58, −18, 47]), as well as thalamus (*xyz* coordinates: N1 = [5, −13, 6]; N2 = [12, −14, 12]). In deep sleep, sources of α/σ activity also encompassed sensorimotor areas (BA 1, 6; *xyz* coordinates: N3 = [−46, −24, 54], [−39, −21, 64]), along with the frontal eye field (BA 8; *xyz* coordinates: N3 = [28, 26, 48]) and parahippocampal region (BA 36; *xyz* coordinates: N3 = [−24, −5, 36]) (Fig. 2*D*).

**Figure 3.**
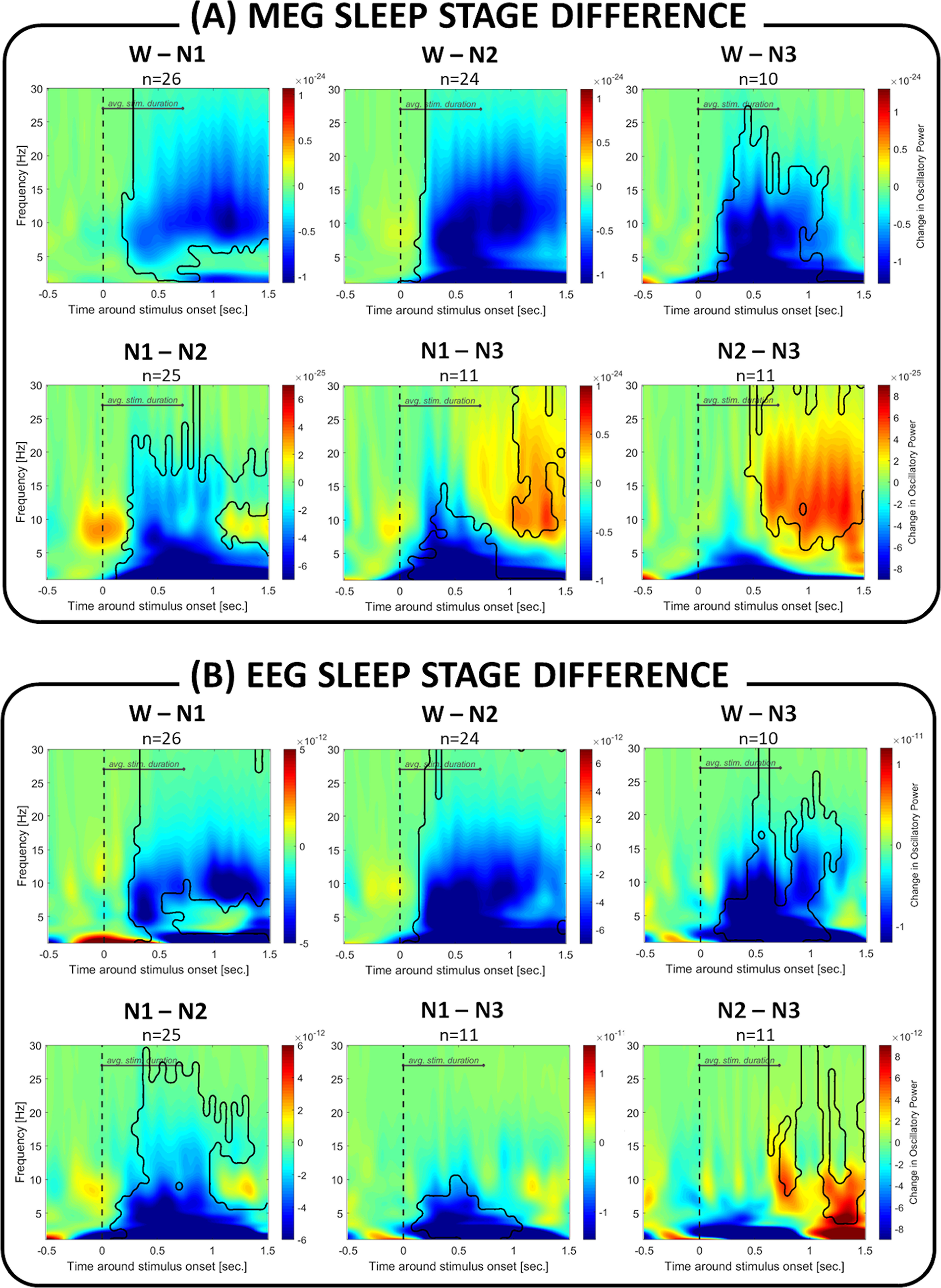
Difference between sleep stages in brain response to auditory stimuli. In (***A***) MEG sensors and (***B***) EEG sensors, the transition from wakefulness to NREM sleep is characterized by significant changes in induced brain responses. The deeper the sleep (N1 to N2 to N3), the stronger the response in the α/σ frequency band (shown in negative values, blue) to auditory stimuli. Additionally, deep N3 sleep was characterized by a weaker response in the late (>50 0 ms) α/σ frequency window compared with N1/N2 (in red). Areas outlined in black represent significant clusters for between-sleep-stage contrasts.

Figure 3 illustrates how the induced brain responses differed between different vigilance states (W to N3) in MEG as well as EEG. Compared with wakefulness, NREM sleep was characterized by a significantly larger response in the α/σ frequency band (W vs N1 in MEG: p_cluster_ < 0.001 and EEG: p_cluster_ < 0.001; W vs N2 in MEG: p_cluster_ < 0.001 and EEG: p_cluster_ < 0.001; W vs N3 in MEG: p_cluster_ = 0.002 and EEG: p_cluster_ < 0.001). A similar effect was observed when we compared drowsiness (N1) to deeper stages of NREM sleep (N1 vs N2 in MEG: p_cluster_ < 0.001 and EEG: p_cluster_ < 0.001; N1 vs N3 in MEG: p_cluster_ = 0.011 and EEG: p_cluster_ = 0.006). Last, comparing NREM sleep stages, we found a significant decrease in deep N3 sleep in a late α/σ frequency band (N1 vs N3 in MEG: p_cluster_ = 0.018; N2 vs N3 in MEG: p_cluster_ < 0.001 and EEG: p_cluster1_ < 0.001, pcluster_2_ = 0.008).

#### Stimulus discrimination

**Figure 4.**
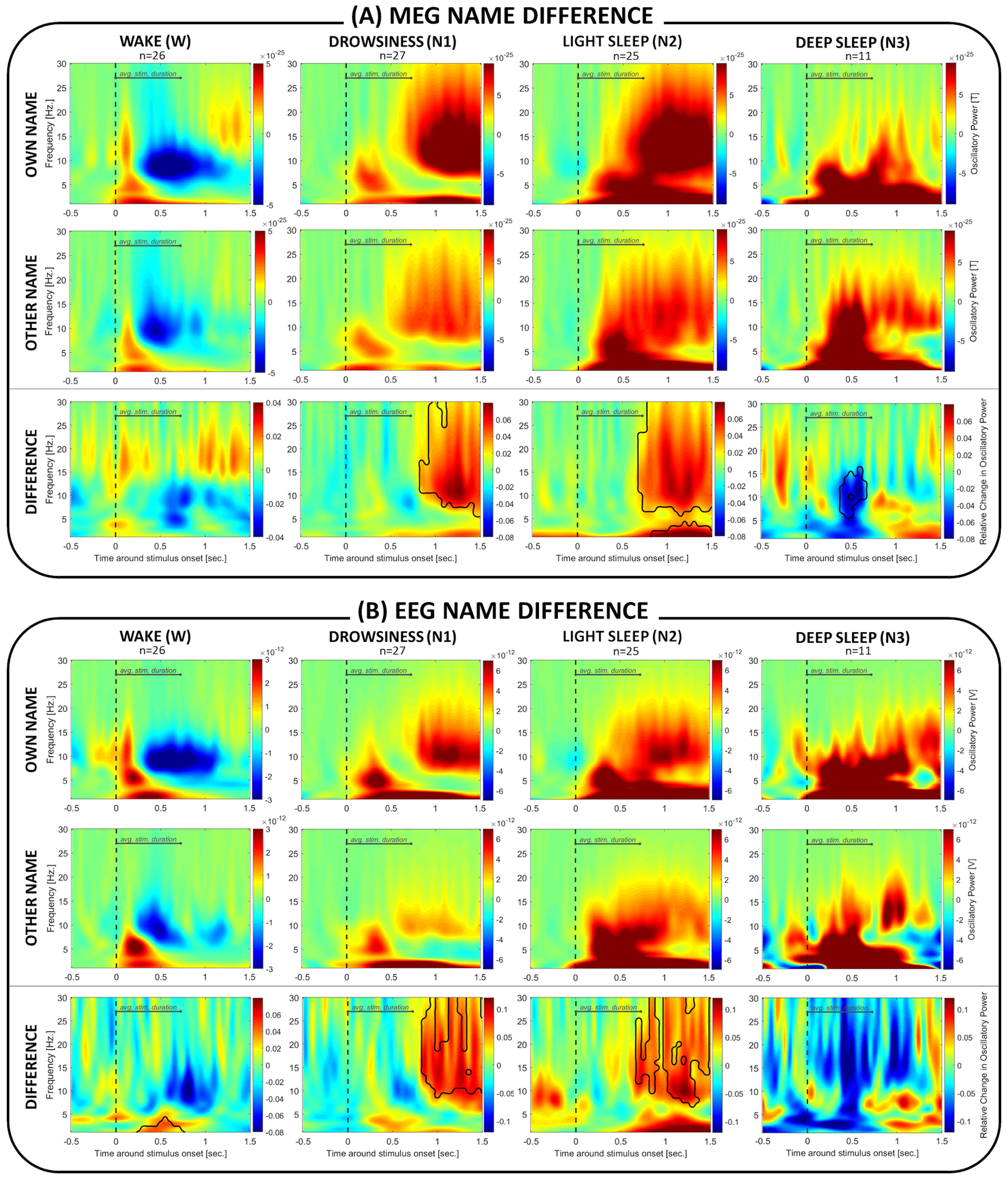
MEG/EEG brain response differences depending on stimulus saliency. Plots represent (***A***) MEG and (***B***) EEG oscillatory brain responses to two types of cues (own vs other names) separately, and the difference between them (subject's own first name *minus* other first name). Interestingly, the brain differentiated between the presented stimuli even during all NREM sleep stages. During N1 and N2, the own name induced stronger responses (in a broad 5-30 Hz oscillatory band). During N3, on the other hand, it was the other name that induced a stronger response in a 5-17 Hz frequency window. Areas outlined in black represent significant clusters. The depicted brain response to own and other names is normalized against absolute baseline between 0.5 and 0.1 s before stimulus onset.

In a next step, we inspected the brain's capacity to discriminate between different stimuli across vigilance states. Figure 4 shows differences in oscillatory response to the names varying in saliency (subject's own vs other first name), as measured by MEG (Fig. 4*A*) and EEG (Fig. 4*B*).

Cluster-based permutation tests run on the MEG signal, revealed a statistically significant effect in all NREM sleep stages (N1: p_cluster_<0.001; N2: frequencies >5 Hz: p_cluster_<0.001; N2 frequencies <5 Hz: p_cluster_ = 0.02; deep sleep) (Fig. 4*A*). In lighter (N1 and N2) sleep stages, the subject's own name compared with other first names induced stronger responses in a late time window (∼1-1.5 s after stimulus onset), across frequencies spanning the α-σ-β range (∼9-30 Hz). Interestingly, in deep sleep, the pattern of brain responses reversed with α/σ showing stronger response after other names compared with own name in an early time window ∼0.5 s after stimulus onset.

Generally, EEG brain responses overlapped with the MEG results and confirmed responses of α-σ in N1 (p_cluster_ = 0.03) and N2 (p_cluster_<0.001) sleep (Fig. 4*B*). During wakefulness, a trend in the same direction was observed (p_cluster_ = 0.08); however, it occurred in an earlier time window (0.3-0.7 s after stimulus onset) and across lower frequencies (∼1-5 Hz). In deep sleep, no significant difference between the names varying in saliency was found in the EEG, although the pattern partly resembled the one seen in MEG.

**Figure 5.**
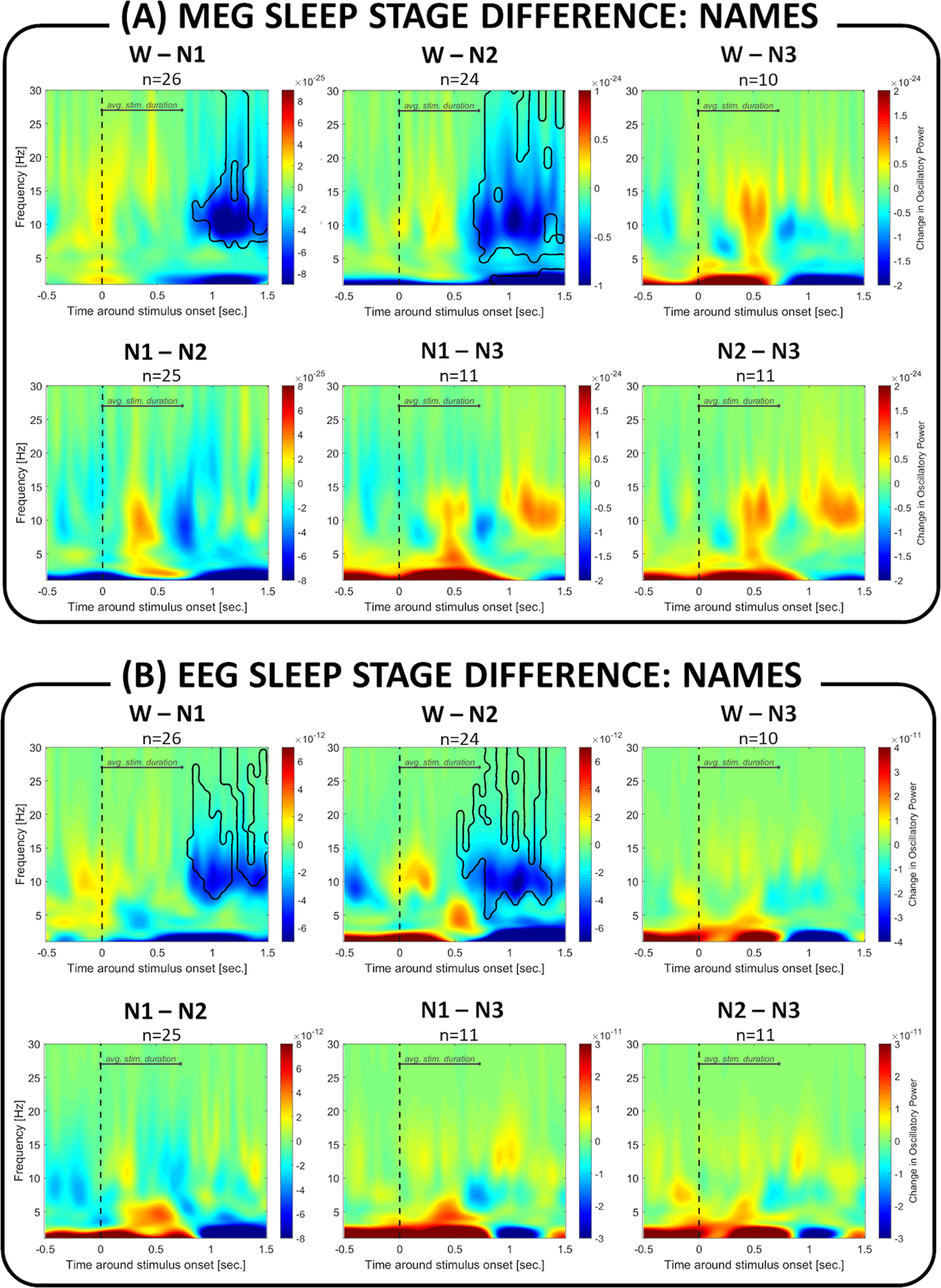
Own versus other name brain response difference between sleep stages. (***A***) MEG as well as (***B***) EEG activity revealed a more prominent α-σ frequency band response during drowsiness (N1) and light sleep (N2) compared with wakefulness (W). Areas outlined in black represent significant clusters for between-sleep-stage contrasts.

In the following, we checked how oscillatory signatures related to name discrimination (own *minus* other name) differed between sleep stages (Fig. 5). The only statistically significant change in brain activity was from wakefulness to both drowsiness (N1, MEG: p_cluster1_ = 0.02, p_cluster2_<0.001; EEG: p_cluster1_ = 0.01, p_cluster2_ = 0.039) and to light sleep (N2, MEG: p_cluster_ = 0.045; EEG: p_cluster_ = 0.04).

**Figure 6.**
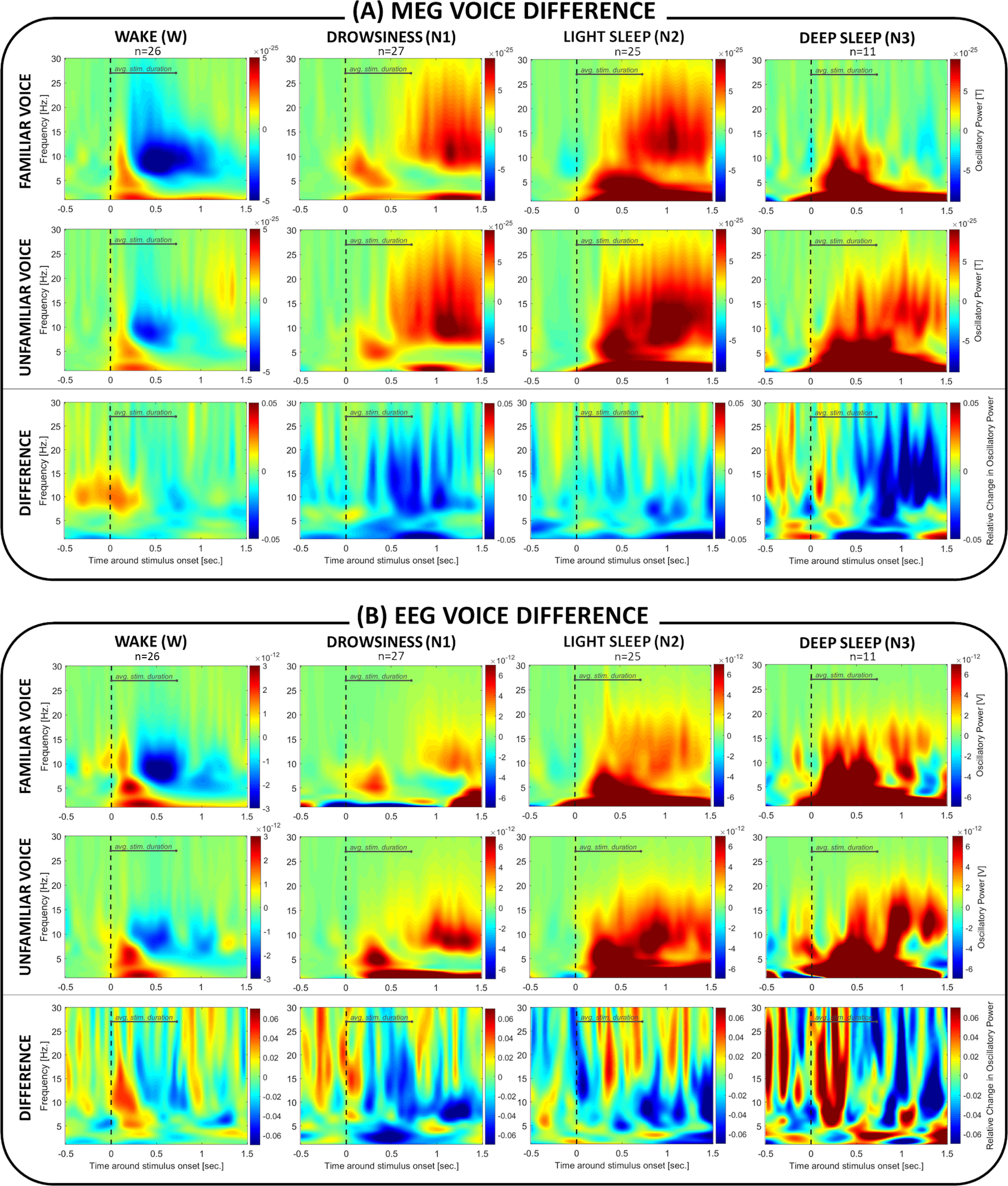
Brain response differences depending on voice familiarity during sleep. Plots represent (***A***) MEG and (***B***) EEG oscillatory brain responses to names uttered by a familiar versus unfamiliar voice and indicate stronger responses to the unfamiliar voice during NREM sleep (which however does not reach statistical significance). The depicted brain response to familiar and unfamiliar voices is normalized against absolute baseline between 0.5 and 0.1 s before stimulus onset. Statistical analysis did not reveal any significantly different brain responses to the two types of voices in either wakefulness or sleep.

The analysis of brain responses to familiar versus unfamiliar voices revealed no statistically significant effects in EEG as well as MEG across states (Fig. 6).

### Sleep spindles have a unique fingerprint

**Figure 7.**
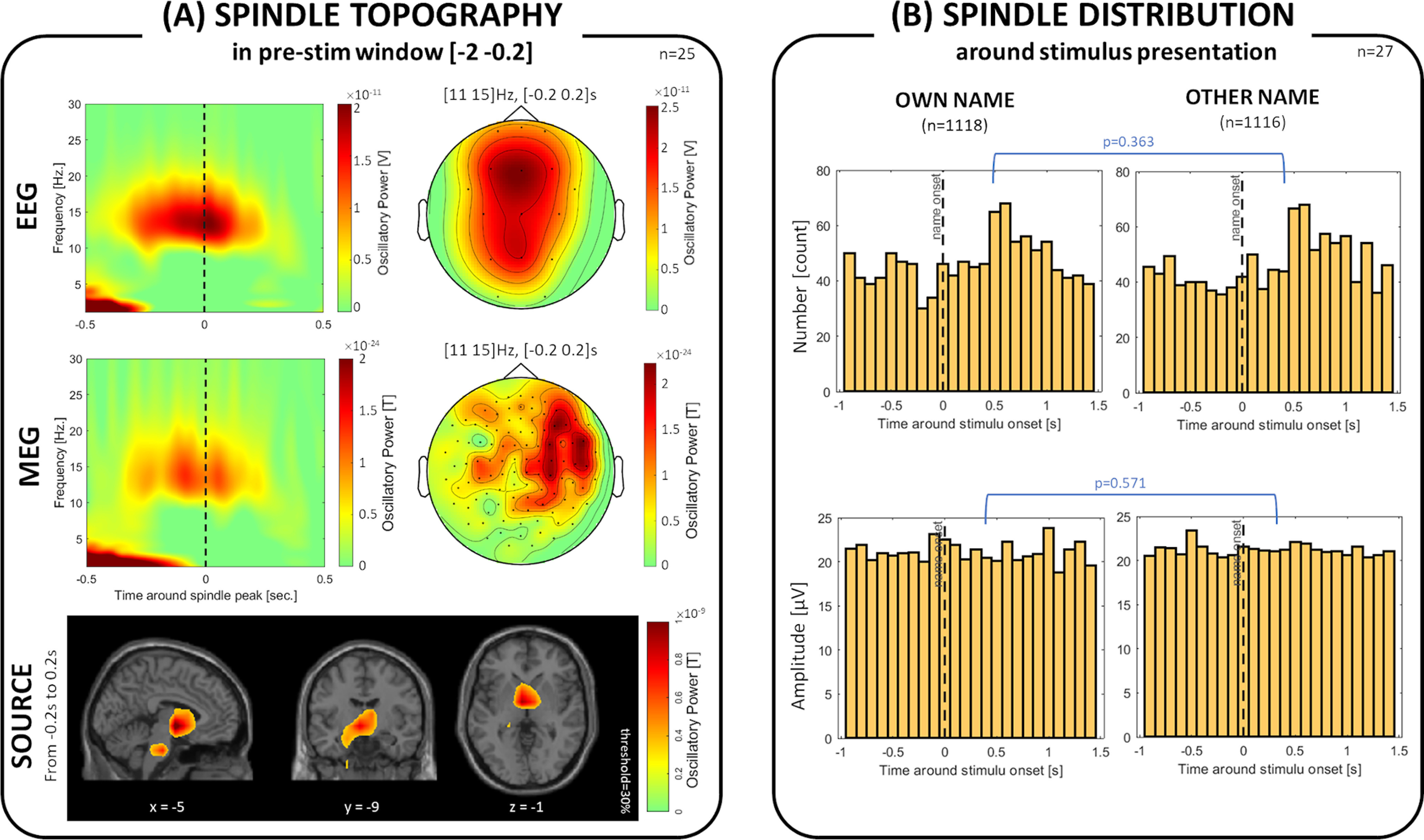
Sleep spindle topography in EEG and MEG. ***A***, Spontaneous sleep spindles detected in the prestimulus time window (−2 to −0.2 s relative to the stimulus onset) peak at ∼14 Hz and have the highest power over medial, frontocentral EEG sensors and lateral MEG sensors. MEG source reconstruction suggests deep sources surrounding thalamus, hippocampus, and pons. ***B***, Histograms of sleep spindle onsets relative to stimulus onsets show accumulations at ∼500 ms after stimulus presentation, which however appears not to be name category-specific. x, y and z coordinates indicate location in MNI space.

Next, we explored the potential contribution of sleep spindles for the observed α/σ frequency responses to the name stimuli. Figure 7*A* depicts spectral and topographical distribution of sleep spindles that emerged spontaneously and in the absence of auditory input during N2 and N3 sleep stages. The source reconstruction encompassed subcortical areas, including (*xyz* coordinates in MNI space): thalamus = [10, −5, 7], hypothalamus = [−5, −3, −7], hippocampus = [−23, −29, −9], parahippocampal gyrus = [−23, −37, −7], amygdala = [−18, −7, −18], and the pons = [−6, −24, −33].

Furthermore, we checked whether sleep spindles changed as a function of specific stimulus characteristics in sleep. Figure 7*B* shows that neither the distribution of spindle number (D = 0.039, *p* = 0.363), nor the distribution of spindle amplitude (D = 0.033, *p* = 0.571), revealed statistically significant difference between own and other names.

### α-σ contributions for the auditory evoked MEG and EEG components from wakefulness to sleep

**Figure 8.**
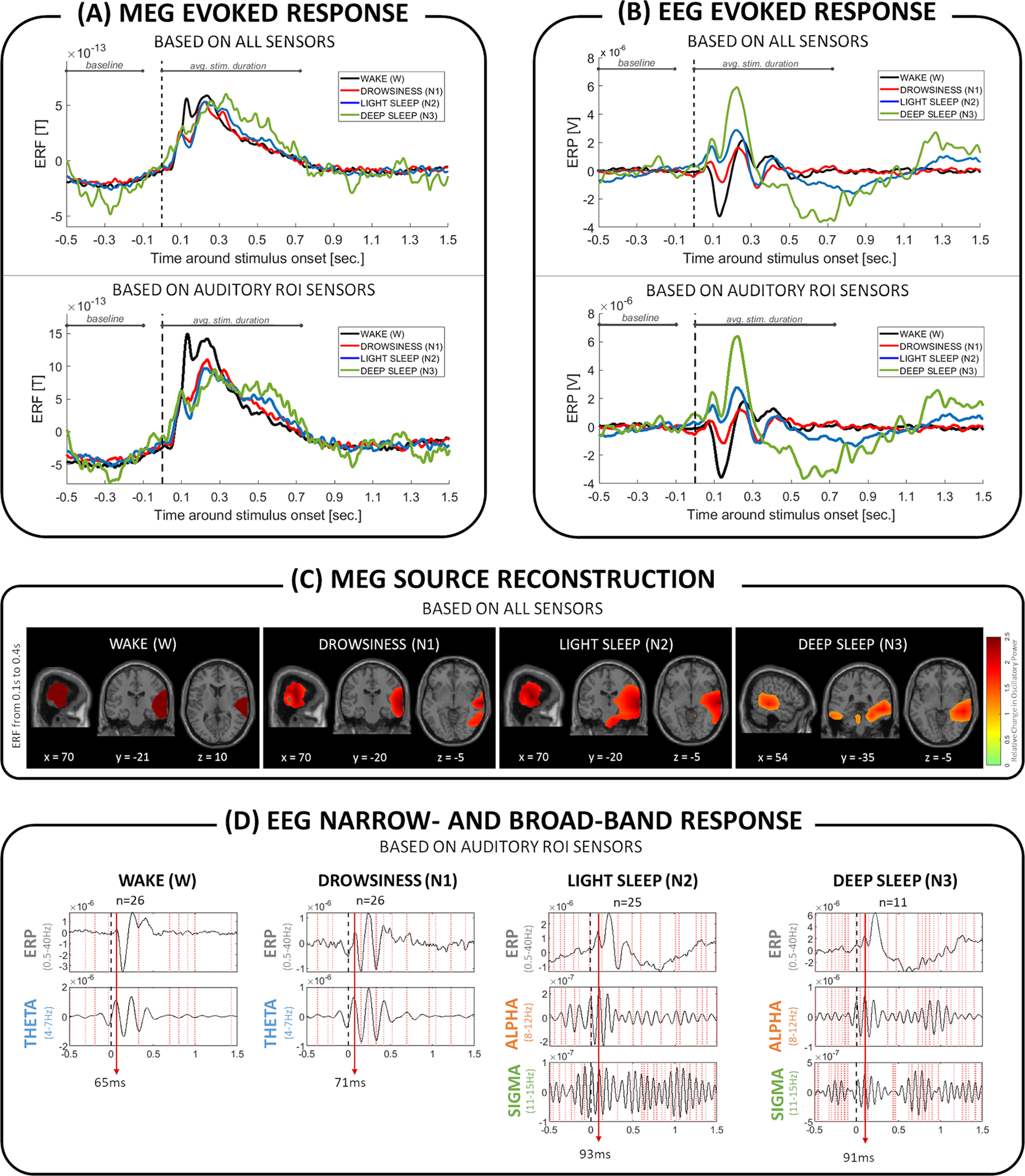
Frequency-specific brain responses evoked by all stimuli. Two top panels represent brain responses evoked by auditory stimuli in wakefulness and the three NREM sleep stages, in (***A***) MEG and (***B***) EEG. ***C***, Source reconstruction of the MEG signal revealed involvement of the primary auditory cortices in the generation of the early (0.1-0.4 s after stimulus onset) evoked responses across wakefulness and NREM sleep. Maps represent the difference between baseline and poststimulus interval, and show the highest 60% of the relative change values, where *x*, *y*, *z* coordinates indicate the location in MNI space. ***D***, The plots compare the appearance of ERP peaks with the narrow-band peaks and troughs in each NREM sleep stage separately. Vertical lines indicate peak-to-peak or trough-to-trough alignment within ±2 sample points. θ oscillations (4-7 Hz) generated the first ERP component during wakefulness and drowsiness. During consolidated NREM sleep (N2 and N3), on the other hand, the first ERP component seems to be generated by faster and phase-synchronized α (8-12 Hz) and σ (11-15 Hz) oscillations.

To further explore the oscillatory mechanisms involved in information processing during NREM sleep, we investigated time-locked activity, that is event-related fields (ERFs) and ERPs across frequencies. As seen in Figure 8, presented names evoked brain responses in wakefulness and NREM sleep, both in MEG (Fig. 8*A*) and EEG (Fig. 8*B*). With the beamformer technique, we then sought for the underlying sources of the observed MEG activity (Fig. 8*C*). Primary auditory cortices along with Wernicke's area likely generated the early ERF field responses (0.1-0.4 s following stimulus onset), independently of the vigilance state (BA 41, 21, 22; *xyz* coordinates of the source level response in the MNI space: W = [70, −21, 10], [65, −21, 4]; N1 = [59, −19, 6], [63, −19, −5]; N2 = [59, −21, 6], [70, −21, −1]; N3 = [56, −29, 10], [54,−35,−4]). During NREM sleep, we observed additional coactivation of the right fusiform gyrus (BA 37; *xyz* coordinates: N1 = [64, −52, −20]; N2 = [61, −45, −19]) and of the pons (*xyz* coordinates: N2 = [10, −22, −38]; N3 = [−6, −25, −34]).

Building on the assumption that ERPs are generated by superimposed oscillations via transient phase alignment (Klimesch et al., 2004, 2007; for an application in sleep research, see Karakaş et al., 2007), we additionally investigated narrow-band-filtered evoked brain responses. Figure 8*D* shows the time alignment of ERP filtered in a broadband frequency (0.5-40 Hz), together with the filtered narrowband frequencies (θ, α, σ) thought to generate the prominent ERP peaks. According to that analysis during wakefulness and drowsiness, the first ERP component was likely generated and dominated by θ (4-7 Hz) oscillations. During consolidated stages of NREM sleep, on the other hand (N2 and N3), the first ERP component was generated by synchronized (phase aligned) and somewhat faster oscillations within the α (8-12 Hz) and σ (11-15 Hz) frequency band (Fig. 8*D*).

## Discussion

By tracking the temporal organization of oscillatory activity, we observed that the brain processes external information depending on the current vigilance level and consequently physiological state. Interestingly, reorganization of the oscillatory activity during NREM sleep did not preclude processing (Fig. 2) or differentiation of acoustic stimuli even at a semantic level (Fig. 4). Oscillatory mechanisms governing these processes during NREM sleep were found to include mainly θ, σ, and α frequency bands. In the following, we will discuss the potential functional role of these oscillations in the sleeping brain and in the light of an auditory processing demand.

θ power increase originating in primary auditory areas, as revealed by MEG source reconstruction, was universally seen in response to acoustic stimulation in wakefulness as well as NREM sleep (Fig. 2). One could relate it to the functional role of θ in episodic memory, which has been described in the awake brain (Klimesch et al., 1994; Doppelmayr et al., 1998; Staudigl and Hanslmayr, 2013). In a similar context, Fellinger et al. (2011) observed θ power increased in response to active counting of own first name presentation, in post-comatose patients. This implies that, even in a state of reduced awareness, stimuli with sufficient bottom-up strength may activate a corresponding episodic memory trace. Unlike α and σ, θ responses continued throughout the wake-sleep spectrum (Fig. 2). It is fascinating that this mode of sensory processing seemingly remains unchanged, independently of the physiological or vigilance state of the brain. In studies investigating mechanisms of sleep-dependent memory reactivation and consolidation, θ was also identified to coordinate reprocessing of memories during sleep and wakefulness (Schreiner et al., 2018). Together, θ oscillations play a crucial role in memory-related processes in the sleeping (in addition to the awake) brain. However, the multifunctional role of θ oscillations during wakefulness (Kahana et al., 2001; Karakaş, 2020), along with limited top-down control of cognitive processing during sleep, renders our interpretation speculative and open for further discussion.

The second dominant brain response to acoustic stimulation involved α and σ oscillations. During light sleep stages (N1 and N2), we mainly observed α to σ power increase after name presentation (Fig. 2), which was stronger in light sleep than in wakefulness (Fig. 3). Furthermore, unlike θ oscillations, α-σ power displayed a prominent difference, depending on whether a subject's own name or another first name was presented (Fig. 4). In more detail, phase-aligned α and σ oscillations appear to initiate the stimulus-processing cascade in consolidated stages of NREM sleep (N2 and N3; Fig. 8*D*). Given that, during NREM sleep, σ becomes the dominant oscillation, we assume that σ drives the transient coupling with α.

By definition, the frequency range of α (∼8-12) and σ (∼11-15) bands overlap (Klimesch, 2012; Buzsáki et al., 2013). It is therefore difficult to completely disentangle these two oscillations, and a possibility remains that our α/σ findings are mainly driven by sleep spindle effects. Given the data, we do not believe so for several reasons: (1) α/σ effects in our study predominantly showed up in N1 and N2 sleep stages, whereas the spindles are dominant in N2 and N3 sleep stages. (2) Sleep spindles were reconstructed to have the highest activity in deep brain regions surrounding thalamus and pons (Fig. 7*A*), while the auditory evoked activity at ∼12-20 Hz had clear cortical peaks (Fig. 2*D*). (3) There was a significant difference in α/σ oscillatory power between own and other names in NREM sleep (Fig. 4*A*), whereas no such difference was evident in the number or amplitude of sleep spindles evoked by the stimuli (Fig. 7*B*). Together, these observations suggest that our α/σ findings presented in Figures 2–5 do not merely reflect sleep spindling activity. It is therefore possible that α and σ frequencies might reflect synchronous activity of distinct neural populations, which perform different functions in processing of external stimuli.

The used stimulus material of first names differed to some degree at the semantic level. Consequently, we speculate that the observed distinct α/σ response to the stimulus category (own vs other first names) reflects varying degrees of semantic information with the own name having an inherent different meaning to the sleeper. The role of σ during NREM sleep might therefore be underestimated and may enable access to stored information specifically in the absence of conscious awareness. Indeed, σ and sleep spindles have been linked to a variety of complex “offline” processes, such as the following: overnight memory consolidation (Cairney et al., 2018), spontaneous (Jegou et al., 2019) and cued (Antony et al., 2018) memory reactivation, acquisition of new associative memories during sleep (Canales-Johnson et al., 2020), as well as thalamocortical and corticocortical connectivity governing information transfer across brain regions (Bonjean et al., 2012). Another possible explanation is that the observed σ power increase is generated by the so-called μ rhythm in somato-motor cortex (Salmelin and Hari, 1994a, b). Indeed, our source reconstruction of σ oscillations revealed a coactivation of the rolandic brain area along with auditory cortex and thalamus, which was specific to (light) sleep (Fig. 2*C*). In that case, σ synchronization could indicate an automatic preparation to or inhibition of a motor response toward the presented stimuli. This is a plausible explanation, especially in the light of recent findings by Andrillon et al. (2016) who observed a preserved lateralized readiness potential in sleeping subjects that were previously instructed to perform semantic categorization with button presses in wakefulness. Timing of our σ effect might give another hint: σ power increase began only after θ power returned to the baseline level, and somewhere around the average stimulus offset.

The role of the α rhythm during NREM sleep is rather elusive. One possibility is that α oscillations, similarly to wakefulness, also during NREM sleep play a transient, yet important, role for accessing the semantic content of the presented stimuli (Klimesch et al., 1997). However, during wakefulness, α power decrease rather than increase characterizes semantic memory processing (Klimesch, 1999). Conversely, several studies described α power increase in paradigms engaging working memory (Klimesch et al., 1999; Jensen et al., 2002; Kaiser et al., 2007), which might in our case indicate sustaining of neural representations of the auditory stimuli (Palva and Palva, 2007). Yet, similar to the interpretation of the θ effect, pinpointing the exact function of the α oscillations during NREM sleep is very challenging, and further research using different sorts of environmental stimuli can help resolving this puzzle. For example, gentle movements of a sleeping person's limbs (similarly to the protocol used by Onishi et al., 2013) might elucidate μ rhythm propagation in different sleep stages worth studying.

Interestingly, the pattern underlying discrimination of stimuli was going in the opposite direction during deep than during light NREM sleep (Fig. 4). A direct statistical comparison, however, failed to reveal significant differences between sleep stages (Fig. 5). Given the limited number of subjects who entered deep sleep in this study (11 of 27), we need to remain careful when interpreting these findings. Noteworthy, a similar deep sleep-specific inversion of the brain response was observed previously in research using continuous speech as stimulus material. Specifically, Legendre et al. (2019) reported that activity of the sleeping subjects' brain preferentially followed an irrelevant story, rather than a simultaneously presented relevant one, but only during deep sleep.

It is surprising that own and other names did not statistically differ during wakefulness in the present study. We can only speculate that this lack of effect reflects habituation and a lack of saliency over the course of repeated stimulus presentation. In total, we had six different stimuli, which were presented repeatedly, without engaging the subject in any active task. The awake brain may therefore neglect or inhibit in-depth processing of information that is considered to be irrelevant. During sleep, on the other hand, we might observe a more sentinel brain response, which cannot be turned off, and which automatically processes information that is potentially relevant or dangerous in an evolutionary sense. Although not significant, we visually also observe the effect of a stronger response to the unfamiliar compared with the familiar voice (Fig. 6), which likewise indicates an automatic focus on unexpected or unfamiliar stimuli during sleep, as recently seen in an hdEEG study of our group (Ameen et al., 2022).

In conclusion, we find strong reorganization of the oscillatory underpinnings of auditory information processing in wake as well as various stages of NREM sleep. This transition likely reflects the function to (1) turn “cognitively inward” while, at the same time, (2) keeping track of potentially relevant external information in the absence of awareness (Andrillon and Kouider, 2020). The main difference between the various vigilance and physiological states lies in the temporal activity profile of various oscillations processing information across all states of vigilance. Although still needing more research, we believe that the current findings are another step toward shedding light on a unified theory of brain rhythms and their functions from wake to sleep.
